# Wavelength-Resolution SAR Ground Scene Prediction Based on Image Stack

**DOI:** 10.3390/s20072008

**Published:** 2020-04-03

**Authors:** Bruna G. Palm, Dimas I. Alves, Mats I. Pettersson, Viet T. Vu, Renato Machado, Renato J. Cintra, Fábio M. Bayer, Patrik Dammert, Hans Hellsten

**Affiliations:** 1 Programa de Pós-Graduação em Estatística, Universidade Federal de Pernambuco, Recife 50670-901, Brazil; 2 Departamento de Engenharia de Telecomunicações, Universidade Federal do Pampa, Alegrete 97546-550, Brazil; dimasalves@unipampa.edu.br; 3 Departamento de Engenharia Elétrica, Universidade Federal de Santa Catarina, Florianópolis 88040-900, Brazil; 4 Department of Mathematics and Natural Sciences, Blekinge Institute of Technology, 371 79 Karlskrona, Sweden; mats.pettersson@bth.se (M.I.P.); viet.thuy.vu@bth.se (V.T.V.); 5 Department of Telecommunications, Aeronautics Institute of Technology (ITA), São José dos Campos, SP 12228-900, Brazil; rmachado@ita.br; 6 Signal Processing Group and Departamento de Estatística, Universidade Federal Pernambuco, Recife 50670-901, Brazil; rjdsc@de.ufpe.br; 7 Department of Electrical and Computer Engineering, University of Calgary, Calgary, AB T2N1N4, Canada; 8 Department of Electrical and Computer Engineering, Florida International University, Miami, FL 33199, USA; 9 Departamento de Estatística and LACESM, Universidade Federal de Santa Maria, Santa Maria 97105-900, Brazil; bayer@ufsm.br; 10 Saab Electronic Defence Systems, 412 76 Gothenburg, Sweden; patrik.dammert@saabgroup.com (P.D.); hans.hellsten@saabgroup.com (H.H.)

**Keywords:** CARABAS II, ground scene prediction, image stack, multi-pass, SAR images

## Abstract

This paper presents five different statistical methods for ground scene prediction (GSP) in wavelength-resolution synthetic aperture radar (SAR) images. The GSP image can be used as a reference image in a change detection algorithm yielding a high probability of detection and low false alarm rate. The predictions are based on image stacks, which are composed of images from the same scene acquired at different instants with the same flight geometry. The considered methods for obtaining the ground scene prediction include (i) autoregressive models; (ii) trimmed mean; (iii) median; (iv) intensity mean; and (v) mean. It is expected that the predicted image presents the true ground scene without change and preserves the ground backscattering pattern. The study indicates that the the median method provided the most accurate representation of the true ground. To show the applicability of the GSP, a change detection algorithm was considered using the median ground scene as a reference image. As a result, the median method displayed the probability of detection of 97% and a false alarm rate of $0.11/{\mathrm{km}}^{2}$, when considering military vehicles concealed in a forest.

## 1. Introduction

Common tasks in synthetic aperture radar (SAR) statistical image processing include the identification and classification of distinct ground type [1,2,3,4,5], modeling [6,7,8,9], and change detection [10,11,12,13]. In special, wavelength-resolution low-frequency SAR systems are useful for natural disasters monitoring, foliage-penetrating applications, and detection of concealed targets [14].

The wavelength-resolution SAR system is usually associated with ultrawideband (UWB) radar signal and ultrawidebeam antenna [15]. With such, the maximum resolution is achieved and it is in the order of radar signal wavelength. Additionally, available UWB SAR systems only operate at low frequencies. One essential feature of wavelength-resolution SAR systems is that the speckle noise does not influence the acquired images since it is likely that only a single scatter is present in the resolution cell. Additionally, small scatterers present in the ground area of interest do not contribute to the backscattering for low-frequency radar systems. Thus, small structures, such as tree branches and leaves, are not shown in SAR images [16]. Because large scatterers are associated with low-frequency components, they tend to be less influenced by environmental effects and are stable in time. Hence, by using multi-passes with identical heading and incidence angle of the illuminating platform at a given ground area, an image package with similar statistics can be obtained [17]. In [18], clutter statistical models for stacks of very-high-frequency (VHF) wavelength-resolution SAR images are discussed. The SAR image stacks are a frequent topic of study for SAR systems with high resolution [19,20,21]. However, the literature lacks the use of large image stacks for wavelength-resolution SAR for change detection applications.

Change detection algorithms (CDA) have been widely considered over the years in the detection of distinct targets in SAR images [22,23,24]. In particular, the wavelength-resolution SAR change detection is an important topic of research and has been studied for more than a decade [17]. Wavelength-resolution systems have also shown unique results with high detectability rate on a low false alarm rate per square km, as presented, for example, in [17,24]. The nature of the wavelength-resolution SAR imagery can be exploited to facilitate the design of CDAs, since (i) the contribution of small scatterers to radar echoes is not significant for the wavelength of several meters; (ii) scatter from large objects are the main contribution; (iii) large scatterers are usually stable in time and less sensitive to environmental effects; and (iv) the wavelength-resolution almost totally cancel the speckle noise [16] in the SAR image given a very stable backscattering between measurements.

A CDA is used to detect changes in a ground scene between distinct measurements in time, such as natural disasters like floods and wildfires or human-made interferences [14]. Generally, in wavelength-resolution systems, a CDA can be simply obtained by the subtraction of two single-look images (reference and surveillance), followed by a thresholding operation. However, an image stack can be considered instead of just two images in a CDA; such a collection of images leads to improved detection performance, as discussed in [17]. This information is used to eliminate clutter and noise in the surveillance image [17], and consequently, enhancing CDA results. Recently, a study using a small stack of multi-pass wavelength-resolution SAR images for change detection was introduced in [17].

In [25], the autoregressive (AR) model was employed as a preliminary study considering a ground scene prediction (GSP) based on a single wavelength-resolution SAR image stack. The resulting predicted image was submitted as input data to a change detection algorithm, based only on subtraction, thresholding, and morphological operations. The CDA in [25] corresponds to the detection analysis step of the CDA used in [26]. Despite its simplicity, the change detection results in [25] were competitive when compared with the ones recently presented in [17,27].

Multi-pass SAR images cannot be exactly equidistantly observed over time since the noise across the image stack is not related to the time order. As a consequence, the use of a time series model, commonly employed in statistical signal processing [28,29,30,31], may not be the most suitable approach to obtain a GSP, and, consequently, resulting in lower performance in a CDA. Additionally, the backscattering of the images in the stack is stable in time, i.e., a sequence of pixels for each position follows a similar pattern, and changes in such behavior are understood as outliers. Thus, an image filtering considering robust statistical methods, such as trimmed mean and median [32,33], might be better candidates to obtain a ground scene prediction. These approaches can provide an accurate prediction of the ground scene, avoid the time order problem, and exclude the pixels that do not follow the sequence pattern. Indeed, the median and the trimmed mean filters are traditionally used to remove impulse noise from an image [34,35,36,37,38,39,40,41].

To the best of our knowledge, the study in [25] is the only work related to the ground scene prediction for wavelength-resolution SAR image stacks. Our paper extends the results presented in [25] with four other statistical methods to predict a ground scene for three SAR image stacks, since statistical methods are commonly employed in SAR image processing [1,2,5,6,7,8,9,10,11,13]. The selected statistical methods to obtain the prediction image are (i) autoregressive models; (ii) trimmed mean; (iii) median; (iv) intensity mean; and (v) mean. The predicted ground scene methods are sought to preserve the ground backscattering statistical characteristics of the images in the stack and presents predicted pixel values closer to the original images. It is expected that the predicted images represent the true ground scenes, allowing applications, such as monitoring of forested areas and natural disasters. In this paper, our goal is twofold. First, we propose the use of statistical methods to obtain a ground scene prediction image based on a wavelength-resolution SAR image stacks. Second, we consider this new image as a reference image in a change detection algorithm. In particular, we employed the median GSP image obtained based on stack statistics as a reference image in a CDA based on the detection analysis step of the CDA presented in [26], which was evaluated in terms of target detection probability and false alarm rate. The results reported in [12,17,24] were adopted as the reference model for comparison.

The paper is organized as follows. In Section 2, we describe the considered change detection method and a suite of selected statistical methods for ground scene prediction. Section 3 presents experimental results, including a description of the considered data set, the ground scene prediction results, and the change detection results. Then, a change detection method based on the discussed GSP approaches is introduced. Finally, Section 4 concludes the paper.

## 2. Change Detection Method

**Figure 1:**
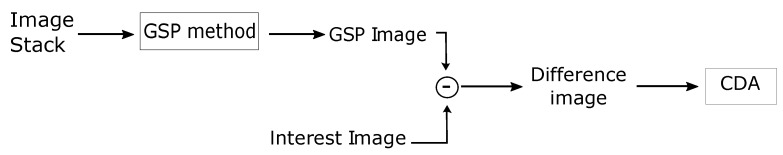
Processing scheme for change detection. The ground scene prediction (GSP) image is the reference image and the interest image is the surveillance image. The change detection algorithm (CDA) is performed applying thresholding and morphological operations in the difference image. Note that the difference image is based on the subtraction between single-look image pixels as a consequence of the stability in backscattering using a wavelength-resolution synthetic aperture radar (SAR) system.

The change detection method used in this paper applied the processing scheme given in Figure 1. An image stack is processed by a desirable GSP method furnishing the GSP image. The changes are simply obtained with the subtraction of the image of interest (surveillance image) from the GSP image (reference image). For change detection, we applied thresholding to the difference image and then used morphological operations for false alarm minimization. The methods employed to obtain the GSP images are described in the next section.

The employed CDA consists of two mathematical morphology steps. First, an opening operation [42] aimed at removing small pixel values, which are regarded as noise. The second step is a dilation that prevents the splitting of the interest targets in multiple substructures. The first step uses a 3×3pixel square structuring element, whose size is determined by the system resolution; the second step considers a 7×7pixel structuring element, which is linked to the approximate size of the targets (about 10×10pixels).

### 2.1. Ground Scene Prediction

**Figure 2:**
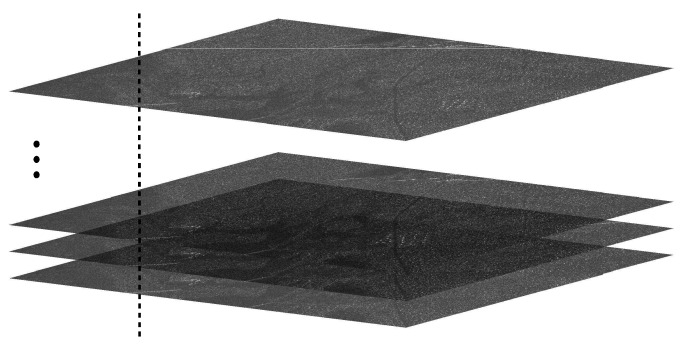
Stack of images to be considered in GSP. The methods should be applied for each pixel position, as evidenced by the vertical line.

As discussed in [18], an image stack is composed of images with similar heading and incidence angle of the same illuminating platform. As a consequence of this similarity, the SAR images in the stack are very similar and stable in time. Thus, a sequence of each pixel position can be extracted from the stack, as illustrated in Figure 2.

The data set considered in this paper is composed of wavelength-resolution SAR images, i.e., the resolution of the SAR image is in the order of the radar signal wavelength [16]. Therefore, there may only be a single scatter in the resolution cell. As a consequence, the considered images are not affected by speckle noise, which is typically a strong source of noise in SAR images in higher frequency bands. Thus, the backscattering from the image stack is stable in time, allowing an accurate GSP.

We consider five statistical methods to obtain ground scene predictions. The techniques are applied in a sequence of pixels, as described in the following.

### 2.2. AR Model

The AR model was adopted to compute the GSP, which can be defined as [43]

(1) $$\begin{array}{c} y\left[n\right]=-\sum_{k=1}^{p}a\left[k\right]y\left[n-k\right]+u\left[n\right],\;n=1,2,\ldots ,N, \end{array}$$

where y[n] is the value of each pixel in one image, *N* is the number of images in the stack, a[k] are the autoregressive terms, u[n] is white noise, and *p* is the order of the model [43]. The autoregressive terms a[k] in Equation (1) can be estimated by the Yule–Walker method [43,44].

Hence, the estimated autoregressive terms $\hat{a}\left[k\right]$ are the solutions of the equation system, given by [43]

(2) $$\begin{array}{c} \begin{array}{c} \left[\begin{array}{cccc} r_{yy}\left[0\right] & r_{yy}\left[1\right] & \ldots & r_{yy}\left[p-1\right]\\ r_{yy}\left[1\right] & r_{yy}\left[0\right] & \ldots & r_{yy}\left[p-2\right]\\ \vdots & \vdots & \ddots & \vdots \\ r_{yy}\left[p-1\right] & r_{yy}\left[p-2\right] & \ldots & r_{yy}\left[0\right] \end{array}\right]\left[\begin{array}{c} a[1]\\ a[2]\\ \vdots \\ a[p] \end{array}\right]=-\left[\begin{array}{c} r_{yy}\left[1\right]\\ r_{yy}\left[2\right]\\ \vdots \\ r_{yy}\left[p\right] \end{array}\right], \end{array} \end{array}$$

where $r_{yy}\left[\cdot \right]$ is the sample autocorrelation function. Information about large sample distributions of the Yule–Walker estimator, order selection, and confidence regions for the coefficients can be found in [45]. Considering the estimated autoregressive terms $\hat{a}\left[k\right]$, it is possible to forecast *h* steps ahead with the AR model as [44]

(3) $$\begin{array}{c} \hat{y}\left[N+h\right]=-\sum_{k=1}^{p}\hat{a}\left[k\right]y\left[N+h-k\right]. \end{array}$$

The ground scene prediction image is obtained by forecasting the one-step ahead (h=1) pixel value for each pixel in the image.

### 2.3. Trimmed Mean, Median, and Mean

For SAR images whose backscattering is stable in time, robust methods can be applied to obtain a GSP. We consider the trimmed mean to obtain a GSP, which is given by

(4) $$\begin{array}{c} {\bar{y}}_{\mathrm{tm}}=\frac{2}{N-2m}\sum_{n=m+1}^{N-m}y^{★}\left[n\right], \end{array}$$

where $y^{★}\left[n\right]$ is the ordered sequence of y[n], m=(N−1)α, and α∈[0,1/2) [32,33]. If α=0 or α→0.5, then the trimmed mean corresponds to the sample mean and median, respectively [32], which are considered as methods for GSP derivation.

### 2.4. Intensity Mean

We also use the intensity mean for obtaining ground scene predictions, given by

(5) $$\begin{array}{c} {\bar{y}}_{\mathrm{im}}=\sqrt{\frac{1}{N}\sum_{n=1}^{N}y{\left[n\right]}^{2}}. \end{array}$$

Compared to other statistical methods, the intensity mean has the advantage of providing physical interpretation about the image reflection. However, the intensities’ values contribute evenly to the prediction results, which can be strongly affected by the changes in the ground scene [32].

## 3. Experimental Results

In this section, we present the results obtained from the discussed ground scene prediction methods and describe an approach for change detection based on such methods.

### 3.1. Data Description

In this study, we considered a data set obtained from CARABAS II, a Swedish UWB VHF SAR system whose images are available in [46]. The system is a low-frequency wavelength-resolution system which means that the images have almost no speckle noise. The data set was divided into three stacks with eight images each, i.e., two out of six passes have identical flight headings. Two passes have a flight heading of $255^{{}^{\circ}}$, two of $135^{{}^{\circ}}$, and two of $230^{{}^{\circ}}$, and the heading is defined as $0^{{}^{\circ}}$ pointing towards the north with clockwise increasing heading. The images in the stacks have the same flight geometry but are associated with four different targets’ deployments (missions 1 to 4) in the ground scene. Hence, with four missions and six passes for each mission, there are 24 magnitude single-look SAR images. The images cover a scene of size 2km×3km and are georeferenced to the Swedish reference system RR92, which can easily be transformed to WGS84 [12,26].

The first stack is composed of images corresponding to flight passes 1 and 3; the second stack, with passes 2 and 4; and the last stack is composed of images associated with passes 5 and 6. In all images, the backscattering was stable in time, and only target changes are expected within the image stacks.

**Figure 3:**
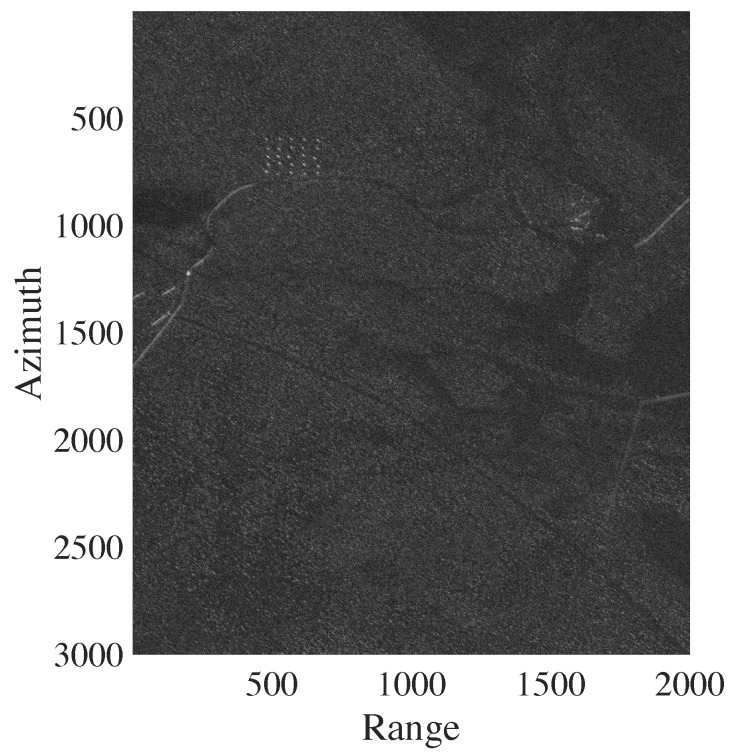
Sample image from CARABAS II data set—Stack 1: mission 1 and pass 1.

Each image is represented as a matrix of 3000×2000 pixels, corresponding to an area of $6\;{\mathrm{km}}^{2}$. As reported in [12], the spatial resolution of CARABAS II is 2.5m in azimuth and 2.5m in range. The ground scene is dominated by boreal forest with pine trees. Fences, power lines, and roads were also present in the scene. Military vehicles were deployed in the SAR scene and placed uniformly, in a manner to facilitate their identifications in the tests [26]. Each image has 25 targets with three different sizes and the spacing between the vehicles was about 50 m. For illustration, one image of Stack 1 is shown in Figure 3. In this image, the vehicles were (i) obscured by foliage; (ii) deployed in the top left of the scene; and (iii) oriented in a southwestern heading. This deployment corresponds to mission 1. In missions 2, 3 and 4, these vehicles were deployed in other locations and were oriented in a northwestern, southwestern, and western heading, respectively [12,26].

### 3.2. Ground Scene Prediction Evaluation

The AR model parameter estimation requires (i) fitting 6,000,000 models (one fit for each pixel) in each stack and (ii) evaluating the best model for each pixel sequence. Such demands lead to a significant computational burden. For simplicity, we considered p=1 in the AR model. Within the image stack, the two images related to the targets have the highest pixel values in the areas where the targets were deployed. Thus, to compute the trimmed mean, we considered m=2(α≈0.3), expecting to remove the pixels related to the targets, since it is desired that the predicted image presents the true ground scene without change.

**Figure 4:**
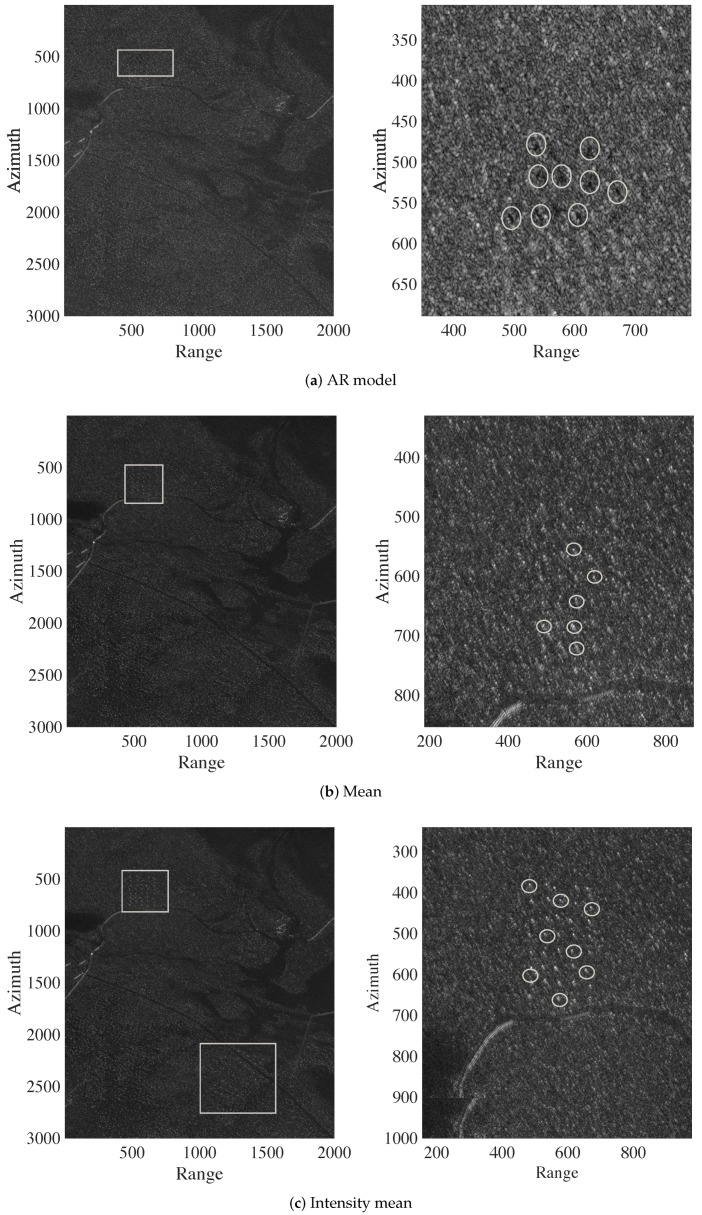
Ground scene prediction images for Stack 1 based on the autoregressive (AR) model, mean, and intensity mean methods. The areas highlighted by rectangles in the images represent the regions where the targets are deployed. The circles show selected military vehicles that can be viewed.

**Figure 5:**
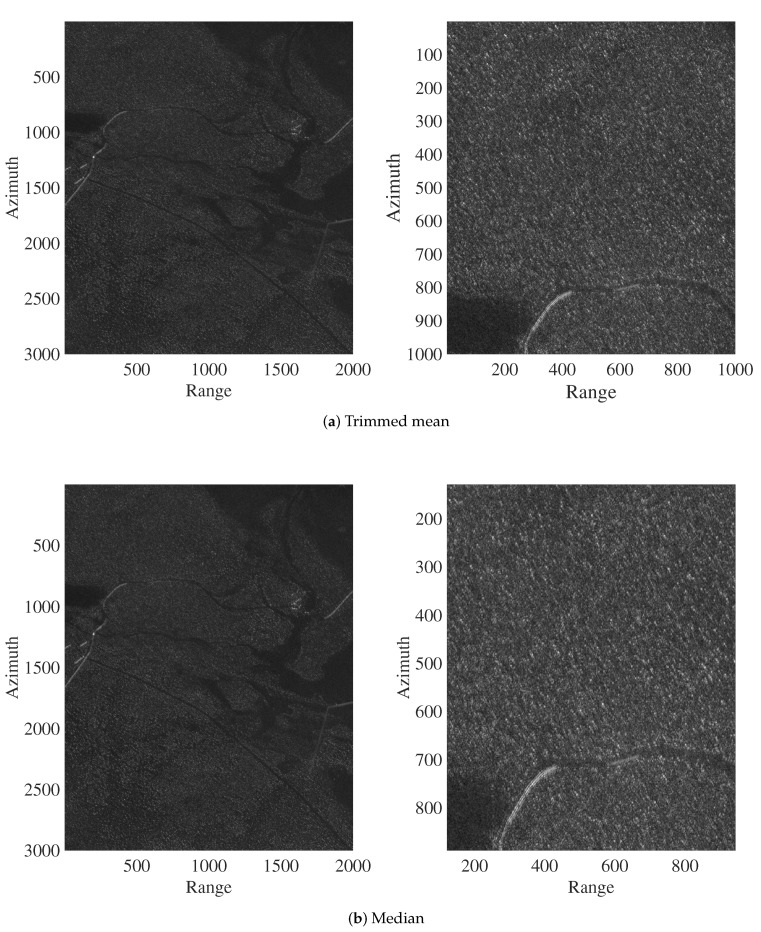
Ground scene prediction images for Stack 1 based on trimmed mean and median methods.

Figure 4 and Figure 5 show the ground scene prediction for Stack 1, considering the discussed methods and a zoomed image in the region where the targets were deployed. In Figure 4, the deployed targets are visually present. However, the targets are absent in the images predicted with the trimmed mean and median, as shown in Figure 5. The areas highlighted by rectangles and circles in the images in Figure 4 indicate the regions where the targets were deployed during the measurement campaign. The circles show selected military vehicles that can be viewed. With such visual analysis, the trimmed mean and median show better performance, i.e., better prediction of the ground scene. For brevity, we limited our presentation to the GSP images from Stack 1, which is representative of all considered stacks.

Table 1 displays descriptive statistics of the employed images, such as average, standard deviation, skewness, and kurtosis. It is desirable that a GSP presents not only a good visual representation of the true ground, but also preserves the statistical characteristics of the image of interest. In Table 1, we highlighted the two best methods according to each considered measure. In the majority of the scenarios, the AR model and median methods outperformed the remaining methods.

To evaluate the difference between the ground scene prediction methods, we computed some standard quality adjustment measures. The criteria are the mean square error (MSE), mean absolute percentage error (MAPE), and median absolute error (MdAE), which can be defined as follows [47].

(6) $$\begin{array}{cc} \mathrm{MSE} & =\frac{1}{Q}\sum_{q=1}^{Q}{\left(x\left[q\right]-\hat{x}\left[q\right]\right)}^{2}, \end{array}$$

(7) $$\begin{array}{cc} \mathrm{MAPE} & =\frac{1}{Q}\sum_{q=1}^{Q}\frac{|x\left[q\right]-\hat{x}\left[q\right]|}{\left|x[q]\right|}, \end{array}$$

(8) $$\begin{array}{cc} \mathrm{MdAE}\; & =\mathrm{Median}\left(|x\left[q\right]-\hat{x}\left[q\right]|\right),\;q=1,2,\ldots ,Q, \end{array}$$

where x[q] and $\hat{x}\left[q\right]$ are the pixel values of the interest and predicted images, respectively, *Q* is the number of pixels, and Median(·) is the median value of $|x\left[q\right]-\hat{x}\left[q\right]|$, for q=1,2,…,Q. These goodness-of-fit measures are usually considered to compare different methods applied to the same data set [47]. They are expected to be as close to zero as possible.

For the quality adjustment measures, the target regions in the image were excluded since we expect to obtain an accurate ground scene prediction, and no target deployment should influence the measurements. Table 2 summarizes the results of the quality adjustment measures for the five considered statistical methods, and the best measurements are highlighted. The mean method presents the best performance according to MSE measurements, while the median method excels in terms of MAPE and MdAE measures in all the stacks. However, the MSE values obtained with the mean and median methods are similar. The results provided in Table 1 and Table 2 consider the same reference image of each stack. Regardless of the selected image, the median method presented good performance according to MAPE, MdAE, and statistics measures.

Based on visual inspection, statistical characteristics, and quality adjustment measures, the median method yields the most reliable prediction among the considered methods. Therefore, we separate the predicted images from the median method as reference images in the change detection algorithm detailed in the next section.

### 3.3. Change Detection Results

**Figure 6:**
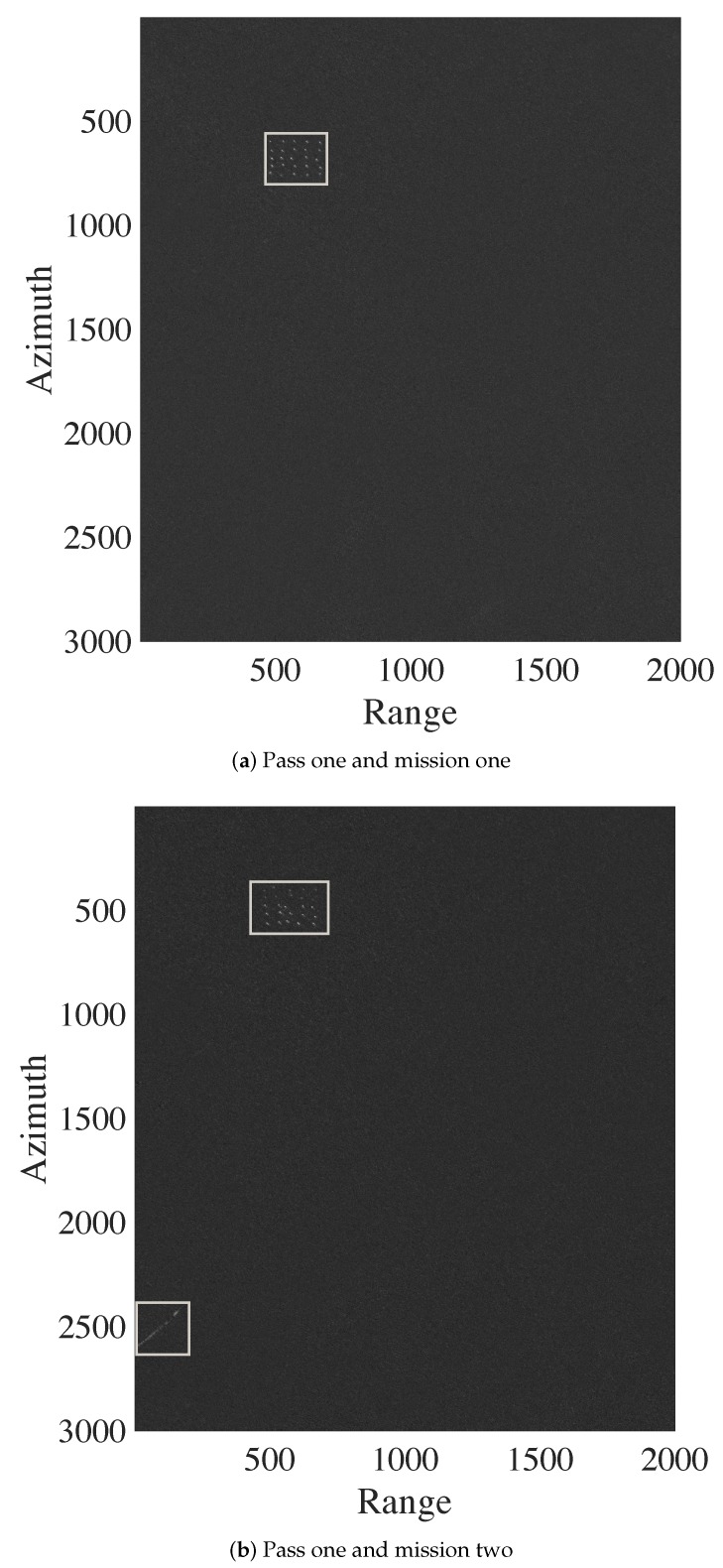
Subtraction of an interest image from the median ground scene prediction image. The areas highlighted by rectangles in the images represent the region with higher pixel values.

As indicated in Figure 1, we use the obtained GSP image and the interest image for change detection based on image subtraction. Two examples of subtraction images are shown in Figure 6. Figure 6a highlights the deployed targets, while Figure 6b focuses on the targets and the back-lobe structures. A comparison between the difference image shown in Figure 6b to the related GSP image suggests that the back-lobe structures are related to issues in the SAR system and the image formation algorithm.

**Figure 7:**
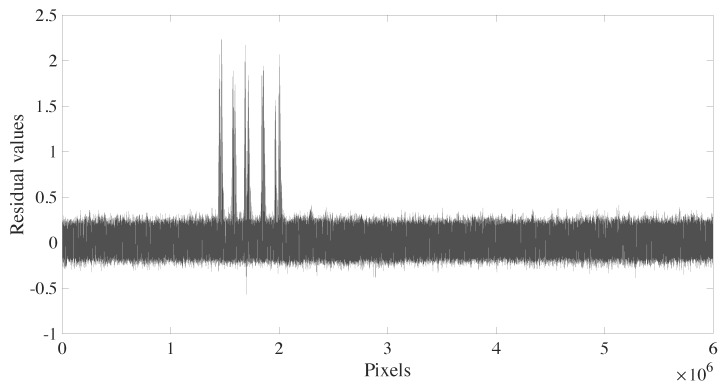
Result of the subtraction of the ground scene prediction image from the image obtained from mission 1 and pass 1.

Figure 7 shows the pixels’ values of the image given in Figure 6a in a vectorized form. In general, the subtracted image pixels values are randomly distributed in (−0.4,0.4). As discussed in [16], the distribution of the values of the CARABAS II subtracted image approximately follows the Gaussian distribution and the regions where no change occurs are stable. Thus, the threshold (λ) can be simply chosen as

(9) $$\begin{array}{c} C=\frac{\lambda -\hat{\mu }}{\hat{\sigma }}, \end{array}$$

where *C* is a constant, $\hat{\mu }$ is the estimated mean, and $\hat{\sigma }$ is the estimated standard deviation of the considered amplitude pixels in the image. For evaluation, we set C∈{2,3,4,5,6}, resulting in different false alarm rates (FAR), which range from full detection to almost null false alarm rate.

Table 3 summarizes the change detection results corresponding to a single constant C=5. Among 600 deployed vehicles in the missions, 579 were correctly detected. There are 22 detected objects that can not be related to any vehicle and are considered to be false alarms. Thus, the detection probability is about 97%, while the false alarm rate is $0.15/{\mathrm{km}}^{2}$ (total of $144/{\mathrm{km}}^{2}$). Ten of the 22 false alarms are related to the back-lobe structures, i.e., they are not actually false alarms and may stem from system and image formation issues. Additionally, in general, the undetected targets are related to missions 2 and 4. These undetected military vehicles are more difficult to detect since they have the smaller sizes and magnitude values, and, consequently, pixel values closer to the forest ones.

### 3.4. Evaluation

**Figure 8:**
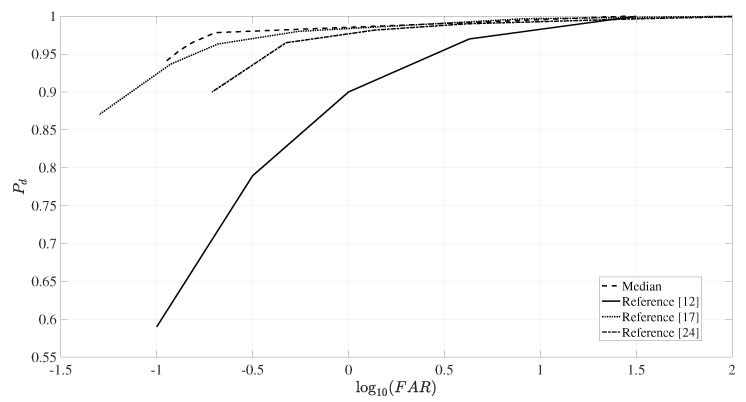
The receiver operating characteristic (ROC) curves obtained with the CDA with the background predicted scene as the reference image compared with the best ROC curves extracted from [12,17,24].

The performance of change detection was evaluated by the probability of detection ($P_{d}$) and FAR. The quantity $P_{d}$ was obtained from the ratio between the number of detected targets and the total numbers of known targets, while FAR is defined by the number of false alarms detected per square kilometer [26]. Figure 8 presents the receiver operating characteristic (ROC) curves [48] of the change detection results, showing the probability of detection versus the false alarm rates for the different evaluated values of *C*. We compared the change detection results obtained from the proposed method with the results described in [12,17,24]. The proposed method excels in terms of probability of detection and false alarm rate in comparison to [12,17,24].

For example, for a detection probability of 98%, our proposed change detection method presents $\log_{10}\left(\mathrm{FAR}\right)$ about −0.5, while [12,17,24] have $\log_{10}\left(\mathrm{FAR}\right)$ about 1.4, −0.3 and 0.14, respectively. For $\log_{10}\left(\mathrm{FAR}\right)=-0.9$, i.e., a very low FAR, the probability detection given by [12] drops to 60%, while our proposal still maintains the probability of detection more than 90%. The detection probability of our proposed method and [17] reach 100% with $\log_{10}\left(\mathrm{FAR}\right)\approx 1$, while [12,24] have full detection for $\log_{10}\left(\mathrm{FAR}\right)\approx 1.5$ and $\log_{10}\left(\mathrm{FAR}\right)\approx 2$, respectively. Additionally, detection probability improvements of our method compared to [17] are found in the range of (0.93,0.98). For example, for a probability of detection of 0.97%, our proposed change detection method presents $\log_{10}\left(\mathrm{FAR}\right)$ about −0.8, while [17] has $\log_{10}\left(\mathrm{FAR}\right)\approx -0.2$.

## 4. Conclusions

In this paper, we presented five methods to obtain ground scene prediction of SAR images based on image stack. The experimental results revealed that, among the considered techniques, the median method yielded the most accurate ground prediction. The statistical characteristics of the obtained GSP image were similar to the image of interest. Moreover, the median method excels in terms of quality adjustment measures, and the changes in the image stack were not visually presented in the predicted image. The GSP image based on the method was used as a reference image in a CDA, presenting competitive performance when compared with recently published results.

## Figures and Tables

**Table 1:** Average, standard deviation, skewness, and kurtosis of one interest image and the ground scene prediction. The interest image in Stacks 1, 2 and 3, is the image of mission 1 and passes 1, 2 and 5, respectively. The two values of each measure that yielded the closest values with the interest image are **highlighted**.

	Average	Standard Deviation	Skewness	Kurtosis
Stack 1				
Interest image	0.1442	0.0894	1.8597	14.1740
AR model	0.1101	0.0725	2.1120	13.5190
Trimmed mean	0.1430	0.0680	2.9051	21.2919
Median	0.1424	0.0688	2.8231	20.4990
Mean	0.1467	0.0663	3.0516	22.8448
Intensity mean	0.1592	0.0667	3.0090	22.8725
Stack 2				
Interest image	0.1373	0.0968	2.9345	30.5666
AR model	0.0997	0.0784	3.6398	40.9991
Trimmed mean	0.1344	0.0806	4.4488	55.4260
Median	0.1339	0.0812	4.3664	53.9367
Mean	0.1376	0.0792	4.6022	58.3558
Intensity mean	0.1485	0.0792	4.5487	57.8894
Stack 3				
Interest image	0.1451	0.0905	1.8583	14.0932
AR model	0.0997	0.0683	2.2034	14.6539
Trimmed mean	0.1372	0.0665	2.8811	22.0954
Median	0.1366	0.0674	2.8090	21.3242
Mean	0.1410	0.0646	2.9582	22.9540
Intensity mean	0.1534	0.0655	2.9170	22.9794

**Table 2:** Measures of quality of the ground scene prediction image. The interest image in Stacks 1, 2 and 3 is the image of mission 1 and passes 1, 2 and 5, respectively. We **highlighted** the values of each quality adjustment measure that yielded the smallest values.

		MSE	MAPE	MdAE
Stack 1	AR model	0.0077	0.6756	0.0548
	Trimmed mean	0.0036	0.6187	0.0364
	Median	0.0037	0.6125	0.0351
	Mean	0.0036	0.6489	0.0401
	Intensity mean	0.0039	0.7505	0.0426
Stack 2	AR model	0.0068	0.6450	0.0502
	Trimmed mean	0.0030	0.5971	0.0326
	Median	0.0031	0.5912	0.0315
	Mean	0.0030	0.6254	0.0359
	Intensity mean	0.0032	0.7204	0.0378
Stack 3	AR model	0.0083	0.6337	0.0557
	Trimmed mean	0.0037	0.5809	0.0357
	Median	0.0038	0.5751	0.0346
	Mean	0.0036	0.6104	0.0392
	Intensity mean	0.0037	0.7011	0.0410

**Table 3:** Change detection results obtained with C=5.

Case of Interest		Number of	Detected	$P_{d}$	Number of
Mission	Pass	Known Targets	Targets		False Alarms
1	1	25	25	1.00	0
2	1	25	25	1.00	3
3	1	25	25	1.00	0
4	1	25	23	0.92	2
1	2	25	25	1.00	0
2	2	25	25	1.00	1
3	2	25	25	1.00	2
4	2	25	23	0.92	1
1	3	25	25	1.00	2
2	3	25	23	0.92	0
3	3	25	25	1.00	3
4	3	25	23	0.92	0
1	4	25	25	1.00	0
2	4	25	25	1.00	0
3	4	25	25	1.00	1
4	4	25	23	0.92	0
1	5	25	25	1.00	0
2	5	25	15	0.60	6
3	5	25	25	1.00	0
4	5	25	24	0.96	0
1	6	25	25	1.00	0
2	6	25	25	1.00	1
3	6	25	25	1.00	0
4	6	25	25	1.00	0
Total		600	579	0.97	22
